# Bortezomib-releasing silica-collagen xerogels for local treatment of osteolytic bone- and minimal residual disease in multiple myeloma

**DOI:** 10.1186/s13045-024-01636-4

**Published:** 2024-12-18

**Authors:** Dirk Hose, Seemun Ray, Sina Rößler, Ulrich Thormann, Reinhard Schnettler, Kim de Veirman, Thaqif El Khassawna, Christian Heiss, Anne Hild, Daniel Zahner, Francisca Alagboso, Anja Henss, Susanne Beck, Martina Emde-Rajaratnam, Jürgen Burhenne, Juliane Bamberger, Eline Menu, Elke de Bruyne, Michael Gelinsky, Marian Kampschulte, Marcus Rohnke, Sabine Wenisch, Karin Vanderkerken, Thomas Hanke, Anja Seckinger, Volker Alt

**Affiliations:** 1 Laboratory of Hematology and Immunology & Labor für Myelomforschung, Vrije Universiteit Brussel, Laarbeeklaan 103, 1090 Jette, Belgium; 2 Experimentelle Unfallchirurgie (ForMED), Justus-Liebig-Universität Gießen, Aulweg 128, 35392 Gießen, Germany; 3 Institut für Werkstoffwissenschaft, Max-Bergmann-Zentrum für Biomaterialien, Technische Universität Dresden, Budapester Straße 27, 01069 Dresden, Germany; 4 Justus-Liebig-Universität Gießen, Ludwigstraße 23, 35392 Gießen, Germany; 5 Klinische Anatomie und Experimentelle Chirurgie C/O Institut für Veterinär-Anatomie, -Histologie und -Embryologie, Justus-Liebig-Universität Gießen, Frankfurter Straße 98, 35392 Gießen, Germany; 6 I. Physikalisches Institut, Justus-Liebig-Universität Gießen, Heinrich-Buff-Ring 16, 35392 Gießen, Germany; 7 Innere Medizin IX - Abteilung für Klinische Pharmakologie und Pharmakoepidemiologie, Medizinische Fakultät/Universitätsklinikum Heidelberg, Im Neuenheimer Feld 410, 69120 Heidelberg, Germany; 8 Labor Für Experimentelle Radiologie, Justus-Liebig-Universität Gießen, Carl-Maria-von-Weber-Straße 8, 35392 Gießen, Germany; 9 Zentrum für Translationale Knochen-, Gelenk- und Weichgewebeforschung, Technische Universität Dresden, Fetscherstraße 74, 01307 Dresden, Germany; 10 Physikalisch-Chemisches Institut, Justus-Liebig-Universität Gießen, Heinrich-Buff-Ring 17, 35392 Gießen, Germany; 11 Klinik und Poliklinik für Unfallchirurgie, Universitätsklinikum Regensburg, Franz-Josef-Strauß-Allee 11, 93053 Regensburg, Germany

**Keywords:** Bone substitute material, Bortezomib, Multiple myeloma, Osteolytic bone lesions, Local treatment, Minimal residual disease

## Abstract

**Background:**

Accumulation of malignant plasma cells in the bone marrow causes lytic bone lesions in 80% of multiple myeloma patients. Frequently fracturing, they are challenging to treat surgically. Myeloma cells surviving treatment in the presumably protective environment of bone lesions impede their healing by continued impact on bone turnover and can explain regular progression of patients without detectable minimal residual disease (MRD). Locally applicable biomaterials could stabilize and foster healing of bone defects, simultaneously delivering anti-cancer compounds at systemically intolerable concentrations, overcoming drug resistance.

**Methods:**

We developed silica-collagen xerogels (sicXer) and bortezomib-releasing silica-collagen xerogels (boXer) for local treatment of osteolytic bone disease and MRD. In vitro and in vivo (tissue sections) release of bortezomib was assessed by ultrahigh-performance liquid chromatography coupled to tandem mass spectrometry (UPLC-MS/MS) and time-of-flight secondary ion mass spectrometry (ToF-SIMS). Material impact on bone formation was assessed in vitro regarding osteoclast/osteoblast numbers and activity. In vivo, drilling defects in a rat- and the 5T33-myeloma mouse model were treated by both materials and assessed by immunohistochemistry, UPLC-MS/MS, µCT, and ToF-SIMS. The material’s anti-myeloma activity was assessed using ten human myeloma cell lines (HMCLs) and eight primary myeloma cell samples including four patients refractory to systemic bortezomib treatment.

**Results:**

sicXer and boXer show primary stability comparable to trabecular bone. Granule size and preparation method tailor degradation as indicated by release of the xerogel components (silica and collagen) and bortezomib into culture medium. In vitro*,* both materials reduce osteoclast activity and do not negatively interfere with osteoblast differentiation and function. The presumed resulting net bone formation with maintained basic remodeling properties was validated in vivo in a rat bone defect model, showing significantly enhanced bone formation for boXer compared to non-treated defects. Both materials induce myeloma cell apoptosis in all HMCLs and primary myeloma cell samples. In the 5T33-myeloma mouse model, both materials stabilized drilling defects and locally controlled malignant plasma cell growth.

**Conclusions:**

The combination of stabilization of fracture-prone lesions, stimulation of bone healing, and anti-tumor effect suggest clinical testing of sicXer and boXer as part of a combined systemic/local treatment strategy in multiple myeloma and non-malignant diseases.

**Supplementary Information:**

The online version contains supplementary material available at 10.1186/s13045-024-01636-4.

## Background

In multiple myeloma, malignant plasma cell accumulation in the bone marrow [1–4] causes lytic bone lesions in 80% of patients during their course of disease. These lesions cause morbidity and mortality, frequently do not heal, and are challenging to treat. After successful systemic treatment, bone lesions are frequently the last sites of visible disease activity [5–7] in magnetic resonance imaging or positron emission tomography. In this protective environment, cancer cells seemingly survive treatment and continue to impede healing by maintained impact on bone turnover. Despite significant improvement of systemic myeloma treatment by introduction of small molecules and immune-oncological drugs by others and us [8–13] and the ability to reduce tumor mass under the detection level (minimal residual disease negativity) [14, 15], ultimately patients relapse and succumb to their disease.

In a 9-year multidisciplinary approach, we aimed at developing bone substitute materials with primary stability comparable to trabecular bone. The material composition should promote healing by stimulating net bone formation, i.e., reducing but not totally blocking osteoclast activity and maintaining or fostering osteoblast function. To tune its biological activity, it should be usable as drug delivery system. For application in myeloma, the material should locally impose tumor control; ideally both by itself and the delivered drugs.

Developed mesoporous silica-collagen xerogels (sicXer) are based, as bone, on mineralized collagen. Xerogels are open networks constructed by removal of all swelling agents from a gel. Everyday examples include structures as gelatin and rubber. Here, silica gel produced from silicon alkoxy compounds by hydrolysis was used. To improve primary stability, silicic acid was chosen for mineralization, inspired by the mechanical strength of marine glass sponge spicules. We modified the silica-collagen ratio to adjust mechanical properties to mimic trabecular bone [16, 17]. We used material and its composition to tailor degradation kinetics and impact on bone remodeling via action on osteoblast and osteoclast development and function by modifying silica-collagen ratio, form of silica [18], and (calcium) phosphate phases [19, 20]. As silica-collagen xerogels consist of non-uniform products harboring disordered free binding sites, ions, and low-molecular organic compounds, they easily absorb [21]. This refers, on one hand, to soluble factors involved in bone remodeling within the implant’s vicinity. On the other, to the payload incorporated in the material prior to implantation. For this, bortezomib was chosen for stimulating net bone formation [22, 23], and being effective and approved for systemic myeloma treatment [24]. Respective materials are termed “boXer”.

We present here the development as well as in vitro and in vivo testing of sicXer and boXer regarding stimulation of bone formation in animal models of normal and myelomatous bone, local myeloma cell control, and ex vivo killing of primary myeloma cells from bortezomib-resistant patients.

## Methods

### Generation and analysis of sicXer and boXer

Production of xerogels was carried out as previously published [17, 25]. Homogeneous suspensions of 30 mg/mL collagen were obtained by dialysis (MWCO 12–14 kDa, Carl Roth) of bovine tropocollagen type I (GfN) against deionized water followed by fibrillation in 30 mM neutral sodium phosphate buffer solution, lyophilization (Christ Alpha1-4 laboratory freeze-dryer), and resuspension in 0.1 M TrisHCl pH 7.4 (Roth) [17]. For sicXer and boXer lyophilized collagen was gamma sterilized at 25 kGy. Silicic acid was prepared by hydrolysis of tetraethoxysilane (TEOS, 99%, Sigma; molar ratio TEOS/water = 1/4) under acidic conditions (0.01 M HCl). Silicic acid and collagen suspension were vigorously stirred by using a vortex mixer to form 800 µL hydrogels with the final composition of 30% collagen and 70% silica. Bortezomib (VELCADE®) was previously added to the collagen suspensions to generate final concentrations of 100 µg (boXer-100), 500 µg (boXer-500), and 2500 µg (boXer-2500) per 1 g silica/collagen xerogel. Hydrogels were stabilized for three days and dried at 37 °C until mass constancy. Monolithic silica/collagen xerogels (diameter: 5 mm, height: 3 mm) were powdered and classified according to suitable particle sizes. siXer-iaf and boXer-iaf (irradiation after fabrication) were gamma sterilized at 25 kGy after fabrication.

#### Sample preparation for degradation studies

For in vitro degradation studies (Supplementary Fig. S1), monoliths of sicXer and sicXer-iaf were incubated in 1.2 mL alpha-MEM medium (αMEM) containing 10% fetal calf serum (FCS), 2 mM glutamine, 100 U/mL penicillin, and 100 µg/mL streptomycin (all from Biochrom). Medium was changed completely three times a week at days 2, 5 and 7. Supernatants were stored at − 20 °C until analysis via inductively coupled plasma mass spectrometry (ICP-MS).

#### Quantification of collagen, silica, calcium, and phosphate

For quantification of collagen in the supernatant, an *ο*-phthaldialdehyd assay was used. In brief, 90 µL of cell culture supernatant was incubated with 100 µL 0.5 mg/mL collagenase from Clostridium histolyticum (Sigma Aldrich) at 37 °C overnight. Then, 20 µL of this were mixed with 200 µL fluoroaldehyd (ThermoScientific) and fluorescence was measured at an excitation and emission wavelength of 340/440 nm (Infinite® M200Pro, Tecan). Different concentrations of bovine collagen in αMEM containing 10% FCS, 2 mM glutamine, 100 U/mL penicillin and 100 µg/mL streptomycin were used for calibration. For quantification of silica, calcium, and phosphate in the supernatant, ICP-MS (IRIS Intrepid II XUV, ThermoFisher Scientific) was used. For analysis preparation, supernatants were diluted in water and HNO_3_ (Carl Roth). Using to a calibration curve measured from element standards (High Purity), ion concentrations were determined.

#### Sample preparation for bortezomib, sicXer and sicXer-iaf release studies

For release studies of bortezomib from boXer-100 and boXer-500, individual fractions of < 125 µm, 125–250 µm and 250–710 µm, were investigated. Here, 9 mg of material were incubated in 700 µL of αMEM containing 10% FCS, 2 mM glutamine, 100 U/mL penicillin and 100 µg/mL streptomycin (all from Biochrom). After 1 h, 2 h, 4 h, 8 h, 24 h, 48 h, 72 h, 96 h, 168 h, 336 h, 504 h, and 672 h supernatants were removed completely and stored at −20 °C until analysis via ultrahigh-performance liquid chromatography coupled to tandem mass spectrometry (UPLC-MS/MS). Quantification of collagen, silica, calcium, and phosphate was performed as described above.

### Impact of sicXer and boXer on in vitro osteoblastogenesis and osteoclastogenesis

#### Osteoblastogenesis

Human mesenchymal stromal cells (hMSC) were isolated from bone marrow aspirates kindly provided by Prof. Bornhäuser et al., Medical Clinic I, Dresden University Hospital. hMSC were expanded in Dulbecco’s modified Eagle’s medium (DMEM), supplemented with 10% FCS, 2 mM glutamine, 100 U/mL penicillin, and 100 µg/mL streptomycin (Biochrom). Osteoblastogenesis on sicXer and sicXer-iaf was investigated. Twenty-four well polystyrene culture plates were seeded with 1.7 × 10^4^ hMSC in passage 5 in 800 µL αMEM containing 10% FCS, 2 mM glutamine, 100 U/mL penicillin, and 100 µg/mL streptomycin. sicXer and boXer granules (15 mg) in ThinCerts (1 µm) were immersed in 1 mL αMEM containing 10% FCS for equilibration overnight. The next day, medium supplemented with 50 µM ascorbate (Sigma Aldrich) was added to the adherent hMSC on their initial day (day 0) in presence of xerogel granules in ThinCert (negative control). For osteogenic differentiation, hMSC were treated with 50 µM ascorbate, 5 mM β-glycerophosphate, and 10 nM dexamethasone (all Sigma Aldrich) by day 3. Medium was changed three times a week. For biochemical analysis (see below), cells were washed twice with phosphate-buffered saline (PBS) and frozen at − 80 °C.

#### Osteoclastogenesis

##### Monocyte preparation

Peripheral blood mononuclear cells (PBMC) were isolated from buffy coats of five healthy donors (German Red Cross). Buffy coats were diluted with equal amount of PBS supplemented with 2 mM ethylendiamintetraacetate (EDTA) and 0.5% bovine serum albumin (BSA; both from Sigma Aldrich) (PBS E/B). Diluted buffy coats were centrifuged for 20 min at 836 g over density gradient 1.077 g/mL (NycoPrep™ 1.077, Progen, Germany) using Leucosep™ tubes (Greiner) without brake. Platelets were removed using density gradient centrifugation over 1.063 g/mL (obtained by dilution of NycoPrep™ 1.077 with PBS E/B) for 15 min at 353 g. Cells were washed with PBS E/B and centrifuged for 8 min at 301 g. Monocytes were isolated from PBMC using negative magnetic separation (Monocyte Isolation Kit II, Miltenyi Biotec) according to the manufacturer’s instructions. Monocytes at a concentration of 1 × 10^7^ were cryo frozen in αMEM containing 10% dimethylsulfoxid (Sigma Aldrich) and 40% heat inactivated FCS until usage.

##### Osteoclastogenesis

Monocytes were thawed and counted (Scepter™, Millipore, Germany). Monocytes (1.5 × 10^5^) in 800 µL αMEM containing 7.5% heat inactivated FCS, 7.5% human A/B serum (ccpro), 2 mM glutamine, 100 U/mL penicillin, and 100 µg/mL streptomycin, additionally supplemented with 50 ng/mL macrophage colony stimulating factor (M-CSF; R&D Systems) were seeded in twenty-four well polystyrene culture plates. sicXer or sicXer-iaf granules (15 mg) in ThinCerts (1 µm, Greiner) were equilibrated overnight using 1 mL αMEM containing 10% heat inactivated FCS. The next day (day 0), by washing twice with PBS, non-attached cells were removed. Moreover, medium supplemented with 25 ng/mL M-CSF and 25 ng/mL receptor activator of nuclear factor kappa B ligand (RANKL; R&D Systems) was added to the adherent monocytes on their initial day (day 0) in presence of xerogel granules in ThinCert. Medium was changed every third day. For biochemical analysis, cells were washed twice with PBS followed by freezing at − 80 °C.

#### Biochemical analysis

Cell lysis was performed with 1% Triton X-100 (Sigma Aldrich) in PBS for 60 min with additional sonication for 10 min. For osteoblastogenesis, activity of lactate dehydrogenase (LDH) and alkaline phosphatase (ALP) was measured from lysates. For osteoclastogenesis, activity of tartrate-resistant acid phosphatase (TRAP) 5b was measured from lysates.

*LDH activity* was used for quantification of hMSC proliferation. Therefore, 50 µL cell lysate was incubated with 50 µL substrate solution of LDH Cytotoxicity Detection Kit (Takara) in the dark. After 15 min, the reaction was stopped using 50 µL 0.5 M HCl (Roth). Absorbance was measured at 492 nm (Infinite® M200Pro, Tecan). LDH activity was correlated with cell number using a calibration curve of cell lysates with defined number of cells.

*ALP activity* was used to quantify osteogenic differentiation, i.e., osteoblast activity. Cell lysates (25 µL) were mixed with 100 µL substrate solution consisting of 5.4 mM 4-nitrophenylphosphate disodium salt in substrate buffer containing 100 mM diethanolamin, 1 mM magnesium chloride, and 0.1% Triton X-100 (all Sigma Aldrich) adjusted to pH 9.8. After incubation for 30 min at 37 °C, reaction was stopped with 50 µL 0.5 M NaOH (Carl Roth). Absorbance was measured at 405 nm (Infinite^®^ M200Pro, Tecan). For calibration, different concentrations of p-nitrophenol (Sigma Aldrich) in substrate buffer were used. ALP activity was normalized regarding cell number assessed via LDH activity.

*TRAP 5b* as osteoclast-specific enzyme was quantified using the substrate naphthol-ASBI phosphate. Cell lysates (10 µL) were added to 50 µL substrate buffer consisting of 2.5 mM naphthol ASBI phosphate (Sigma Aldrich) in 100 mM sodium acetate (Carl Roth), 50 mM sodium tartrate, 2% Nonidet™ NP 40, and 1% ethylene glycol monomethyl ether (all Fluka) adjusted to pH 6.1. The reaction was stopped with 125 µL 0.1 M NaOH (Roth) after incubation for 30 min at 37 °C. Fluorescence was measured at an excitation and emission wavelength of 335/405 nm (Infinite^®^ M200Pro, Tecan). Different TRAP concentrations (BoneTRAP) were used for calibration.

#### Scanning electron microscopy

Samples were prepared on aluminum stubs and coated with carbon. An ESEM XL 30 scanning electron microscope (Philips) working at 3 kV and detecting secondary electrons was used for imaging.

### Impact of sicXer and boXer on primary myeloma cells and myeloma cell lines

#### Patients and samples

Consecutive patients (n = 8) with previously untreated, therapy-requiring (due to the presence of myeloma-defining CRAB-features [26]) or relapsed multiple myeloma were included in the study approved by the ethics committee of the Medical Faculty of the University of Heidelberg (#S-152/2010) after written informed consent.

Myeloma cells were purified from bone marrow aspirates by using anti-CD138 microbeads and an AutoMACS Pro Separator (Miltenyi Biotec); quality control was performed using flow cytometry [9, 27, 28]. The human myeloma cell lines AMO-1, KARPAS-620, KMS-11, KMS-12-BM, L363, OPM-2, RPMI-8226, and U266 were purchased from the German Collection of Microorganisms and Cell Cultures, American Type Cell Culture, or Japan Health Science Research Resources Bank; the HG-lines HG1 and HG9 were generated at the Myeloma Research Laboratory Heidelberg (Germany). Cell line identity was assessed by DNA-fingerprinting, mycoplasma-contamination excluded by PCR-based assays, and EBV-infection status by clinical routine PCR-based diagnostics.

#### Killing of myeloma cell lines

After 24 h pre-incubation of xerogels, myeloma cell lines were exposed to sicXer-iaf, boXer-20-iaf, boXer-100-iaf, and boXer-500-iaf, respectively, and cultured for 72 h. Cell viability was assessed by using a WST-1 based colorimetric assay (Roche) and referred to the medium control without xerogels. Each dose point was done in triplicates.

#### Survival of primary myeloma cells

Primary myeloma cells cultured together with their bone marrow microenvironment of eight myeloma patients were exposed to sicXer-iaf and boXer-500-iaf, respectively. After six days, cell viability was measured by CD138-FITC (IQ products, clone B-A38)/propidium iodide (PI; Pharmingen) staining and referred to the medium control as published [9, 27, 28]. In brief, remaining viable myeloma cells are identified as CD138^+^/PI^−^, remaining viable cells of the bone marrow microenvironment as CD138^−^/PI^−^ cells. Each dose point was done in duplicates. Data analysis was performed using FACSDiva software (BD Biosciences).

### Rat model (drill hole defect) bone healing

sicXer-iaf and boXer-100/500/2500-iaf (particle sizes: 250–710 µm) were implanted into a drill hole defect in the left femur of healthy female Sprague Dawley rats ([Crl:CD(SD)], age of 4 month; Charles River). Animal housing and experimental procedures were performed in full compliance with the institutional and German protection laws after approval by the local animal welfare committee (reference number: V54-19 c20/15-F 31/38). After general anesthesia, the distal femur was approached anterolaterally, and a drill hole defect was made in the metaphyseal area with a diameter of 2.5 mm and 4 mm depth. Post-operatively, animals were individually housed with free access to feed and water. Four weeks post-surgery, animals were euthanized under inhalation of CO_2_ after general anesthesia. Both operated and contralateral femora of each group were harvested and processed as mentioned below. For determination of the bortezomib release, samples from the drill hole itself, the surrounding muscle, and bone samples from the surrounding femoral condyle bone, the distal shaft region and the proximal shaft region were taken.

### Histology and histomorphometry

#### Assessment of new bone formation

Harvested rat femora were fixed in phosphate-buffered 4% paraformaldehyde and stored at 4 °C until processing. Samples were then embedded in Technovit^®^ 9100 NEU according to the manufacturers protocol (HeraeusKulzer). After embedding, Technovit blocks were sectioned into 5 µm thick slices with the aid of Kawamoto´s film (Section-Lab) to prevent the loss of any biomaterial. While sectioning, the plane with both condyles being visible was maintained for all groups.

#### Histomorphometric analyses

Undecalcified bone sections stained with movat pentachrome and toluidine blue staining were used for qualitative and quantitative morphological analyses. Images were obtained with a 10 × objective using a light microscope (Axioplan 2 imaging with photomodule Axiophot 2, Carl Zeiss) and a Leica DC500 camera, acquired with Leica IM1000 software and processed using Adobe Photoshop version CS6.

For histomorphometric analysis of new bone formation (bone volume/trabecular volume; BV/TV) Adobe Photoshop CS6 was used. A single region of interest (ROI) comprising the initially created drill hole defect was chosen. This included both the new bone formation in the former created defect zone and the biomaterial-tissue interface. The ROI was kept constant for all groups. The measurements for ROIs, area of bone, implant, osteoid, and the void were considered to determine the extent of new bone formation.

Osteoblasts were traced on toluidine blue stained slides as blue cuboidal cells aligned in clusters at the bone surface. The osteoblast surface over bone surface (Ob.S/BS) was then determined by tracing directly on the osteoblast cells. The measurements were done blind folded with regards to the test groups.

#### Enzyme histochemical analysis

TRAP staining was used to investigate osteoclast activity. Samples were deplastified, followed by treatment with sodium acetate buffer and incubation in Napthol-AS-TR phosphate in *N*-*N*-dimethyl formamide (both from Sigma Aldrich) and sodium tartrate (Merck) with Fast Red TR salt (Sigma Aldrich) at 37 °C for 1 h.

Counterstaining was done with hematoxylin (Shandon). A count of TRAP-positive cells (osteoclasts) was done in the fixed ROI to determine the osteoclast count per trabecular area (Oc./Tb. Ar).

#### Immunohistochemistry and immune histomorphometry

The following antibodies were used: rabbit anti-BMP-2 polyclonal antibody (AP20597PU-N; Acris), rabbit anti-osteoprotegerin (OPG) polyclonal antibody (250,800; Abbiotec, USA), rabbit anti-CD254/RANKL polyclonal antibody (AP30826PU-N; Acris), rabbit anti-CD31 antibody (250,590; Abbiotec), and mouse anti-rat monocytes/macrophages monoclonal antibody ED1 (MAB1435; Chemicon), respectively.

Goat anti-rabbit (BA-1000, Vector) served as secondary antibody for BMP-2, OPG and RANKL followed by Vectastain ABC kit (Elite PK-6100, Standard, Vector Laboratories). Final visualization was done using Nova Red (SK4800, Vector Laboratories) and hematoxylin (Shandon) was used for counterstaining. ED1 antigen identification was done using DakoEnvision + System-HRP (DAB) for use with mouse primary antibodies (Dako, K4006).

Images were taken using Axioplan 2 Imaging system (Carl Zeiss) with a Leica DC500 camera, acquired with Leica IM1000 software and processed using Adobe Photoshop CS6.

Histomorphometrical analysis for OPG/RANKL ratio [%] was determined by manual count of stained cells for OPG and RANKL. ED1 counts were performed analogously.

### 5T33-mouse model—bone healing and control of myeloma cell proliferation

#### 5T33-mouse model

Animal housing and experimental procedures were realized according to the Institutional Animal Care and Use Committee of the Vrije Universiteit Brussels (license number. LA1230281) and all procedures were approved by the Ethics Committee for Animal Experiments (#15–281-6). sicXer (n = 8) and boXer-500/−2500 (n = 10 each), with particle sizes < 125 µm, were implanted into a drill hole defect of 1 mm diameter and 1 mm depth (see Fig. 4D) in the left femur of the 5T33-myeloma mouse model [29, 30] after anesthesia with Ketamine 100 mg/kg and Xylazine 10 mg/kg (both intraperitoneally). Meloxicam 1 mg/kg was administered subcutaneously for pain control. Mice were sacrificed three weeks after surgery and femora were fixed in 4% paraformaldehyde; one femur of each group was fixed in yellow fix, another one in dry ice for further analyses.

#### Histology and immunohistochemistry

The femoral condyles were fixed in 4% paraformaldehyd (Carl Roth) and embedded in Technovit 9100 (HeraeusKulzer) for histological and immunohistochemical examinations according to the manufacturer protocol.

All condyles were processed sagittally in sections of 4 µm thickness by using a Rotary Microtome (RM 2265, Leica Microsystems).

Masson Goldner staining was performed according to the manufacturer protocol (Carl Roth).

For CD138 immunohistochemistry, sections were deacrylated for 2 × 15 min in 2-methoxyethyl acetate (Merck), hydrated with decreasing alcohol concentration, heat treated at 90 °C with 0.1 M citrate buffer for 20 min, blocked 5 min in 3% H_2_O_2_, and incubated over night at 4 °C with the primary antibody against human, rat, mouse and hamster CD138 (Acris, PAB 9567) diluted 1:1000 in antibody diluent (Dako, DK, S3022). Then, sections were treated 2 × 20 min with SuperVison 2 Single Species HRP-Polymere Rabbit (DCS, PD 000POL-K), subsequently incubated with DAB (DCS, DC 137 C 100) for 10 min, and finally covered with DePeX (Serva).

#### Histomorphometry

All sections were photographed with a 5 × objective by using a Leica DFC 320 mounted on a Zeiss Axiophot. The sections stained by means of Masson Goldner were used to mark and measure the defect area and to calculate the amount of osteoid localized in close vicinity to the defect area. For this purpose, an area of interest was defined by using the Lasso of Adobe Photoshop CS6 Extended including the defect area itself and an area of 100 µm around the defect.

By using the Magic Wand Tool in Adobe Photoshop CS6 Extended, red stained areas of osteoid were marked and measured in mm^2^.

For detection of CD138 positive cells within the bone marrow, a ROI was defined by using the Lasso of Adobe Photoshop CS6 Extended. This area covered the bone marrow within the distal epiphysis around the defects and additionally, the subsequent area of the bone marrow extending 2 mm into the distal diaphysis. By using the Magic Wand Tool in Adobe Photoshop CS6 Extended, the respective area of membrane associated CD138 immunostaining was collected, and the proportional coverage of CD138 positive areas was calculated.

#### UPLC-MS/MS analysis

Bortezomib was determined with UPLC/MS/MS after liquid–liquid extraction as previously described [31]. For ex vivo analysis, explanted tissues (rat model) were homogenized in hydrochloric acid (500 µL, 0.1 M) using an ULTRA-TURRAX (IKA) and subsequently centrifuged (400 × g, 5 min, 4 °C). For liquid–liquid extraction, internal standard solution (25 µL) was added to each sample followed by vortexing (10 s) and sonication (3 min). After addition of 5 mL methyl tert-butyl ether, samples were automatically shaken (overhead, 15 min) and centrifuged (3000 × g, 10 min). The obtained supernatant was evaporated under a stream of nitrogen and reconstituted in 250 µL ACN/H_2_O (30/70, v/v + 0.01% formic acid).

Aliquots of 20 µL were injected into the UPLC-MS/MS system which consisted of an Acquity UPLC System (Waters Sample Manager and Binary Solvent Manager) and a triple stage quadrupole mass spectrometer (Waters Xevo TQ-S). A Waters Acquity BEH C18 column (1.7 μm, 2.1 × 50 mm) with an integrated filter disc was used for chromatographic separation. The acidified eluent (0.01% formic acid) consisted of acetonitrile and H_2_O at a flow rate of 0.8 mL/min. Using positive electrospray ionization, the mass spectrometer detected [M + H]^+^ ions at m/z 367.1 (bortezomib) and m/z 375.2 (D8-bortezomib) via the first quadrupole filter (Q1). After collision-induced fragmentation in argon gas (Q2), product ions were monitored via Q3 at m/z 225.9 (bortezomib) and m/z 233.8 (D8-bortezomib).

For calibration and quality control (QC), blank tissue was spiked with 25 µL of the respective calibration or QC solution resulting in ten calibration standards (0.5–2500 pg per sample) and three QC samples (1.65, 472, and 708 pg per sample) in duplicates per analytical run. Peak area ratios obtained from monitored ions were utilized for construction of calibration curves using weighted (1/x) linear least squares regression. Resulting calibration curves always revealed correlation coefficients greater than 0.998 and allowed a lower limit of quantification of 0.5 pg per sample. In accordance with FDA and EMA guidelines [32, 33], within-batch and batch-to-batch accuracies of the QC samples were within the accepted range (± 15%). Data collection, peak integration, and calculations were performed using Waters TargetLynx V4.1 software (Waters).

### Micro-CT analysis of xerogel-bearing mice femora

Technovit embedded samples were imaged using the μCT system SkyScan 1173 (Bruker MicroCT). Raw data acquisition, image reconstruction and post processing steps were done following the guidelines for assessment of bone microstructure [34]. Scanning parameters were set as follows: tube current: 200 μA, tube voltage: 40 kVp, rotation step width 0.24°, frame averaging for noise reduction: n = 4. For beam filtration, a 0.5 mm aluminum filter was used. NRecon-Software (Bruker microCT) with a gaussian filter was utilized for Image reconstruction of cross sections with an isotropic voxel size of 5.7 μm.

To quantify new bone formation, a layer package of 0.5 mm thickness was defined in the mid-sagittal plane in perpendicular alignment to the drill hole. Within this volume, the volume (i.e., degradation) of the implant, the new bone formation within the drill hole, and the bone mass around the drill hole were determined based on threshold values. The method was adapted to previously reported techniques [35, 36].

For morphometric analysis, binarization of the gray-valued μCT data was carried out using a locally adaptive thresholding technique (CTAn-Software, Bruker microCT).

Following the suggestions made by Bouxsein et al., treatment induced changes in bone morphology were quantified by relative bone volume, trabecular thickness and trabecular number.

### ToF–SIMS analysis

Time-of-flight secondary ion mass spectrometry (ToF–SIMS) enables the simultaneous analysis of organic and inorganic compounds of a sample surface with high mass and high lateral resolution (down to 100 nm). To obtain the chemical information, the sample surface is bombarded with a primary ion beam which impact leads to the emission of secondary ions. These secondary ions are analyzed by a time-of-flight analyzer and separated by their mass to charge ratio. By scanning the primary ion beam over the sample surface, the lateral distribution of the chemical compounds is obtained, and mass landscapes can be created. ToF–SIMS was used here for analysis of bone cross-sections regarding bone quality, degradation of implanted material, and bortezomib release. For detailed methodology and its application in bone research, see Kern et al. [37]. For SIMS-measurements, a ToF-SIMS 5–100 machine (ION-TOF Company) equipped with a 25 keV Bi-cluster ion gun for surface analysis, and an Ar gas cluster ion source as sputter gun was used. Bi_3_^+^ was used as primary ion species. The primary ion gun was operated in spectrometry mode with highest mass resolution and a lateral resolution of about 10 µm. Data evaluation was done with the Surface Lab 6.3 software of ION-TOF Company. During sample preparation, the surface was covered with a layer of resin. This was cleaned with Ar-clusters prior analysis. Therefore, a 10 keV Ar_1500_^+^ cluster beam scanned over the bone surface with a cleaning speed of 0.010–0.025 mm/s for 2–3 times depending on the thickness of the resin layer.

For imaging of rat femora, single images of the size 500 × 500 µm^2^ were stitched together to obtain areas of several square millimeters (so called “stage scans”). For bortezomib detection in boXer group, stage scans were taken with pixel density of 100/mm, cycle time of 50 µs, 500 shots/pixel and 5 patches with three scans resulting in 7500 shots/pixel in total. Empty defects and sicXer group were analysed applying the same conditions using 1 scan to reduce measurement time. Here, the obtained count rates are multiplied with 3 for comparison.

### Statistical analysis

Computations were performed using R 3.1.1 (http://www.r-project.org/) and Bioconductor 2.14 [38]. Effects were considered statistically significant if the *P* value of corresponding statistical test was < 5%. If not otherwise stated, results were expressed as means ± standard deviation. For experiments related to cell culture and bortezomib release, statistical significance was evaluated by analysis of variance (two-way ANOVA, Bonferroni correction for multiple testing, GraphPad Prism). Statistical analysis of immunohistochemistry data was conducted using IBM SPSS Statistics (Version 28.0). Due to the non-normal distribution of the data, non-parametric methods were applied. Differences between treatment groups were assessed using the Kruskal–Wallis H test, followed by pairwise comparisons with the Mann–Whitney U test for significant results.

## Results

### Development and synthesis of (bortezomib-releasing) mesoporous sol–gel silicafibrillar collagen xerogels

**Fig. 1:**
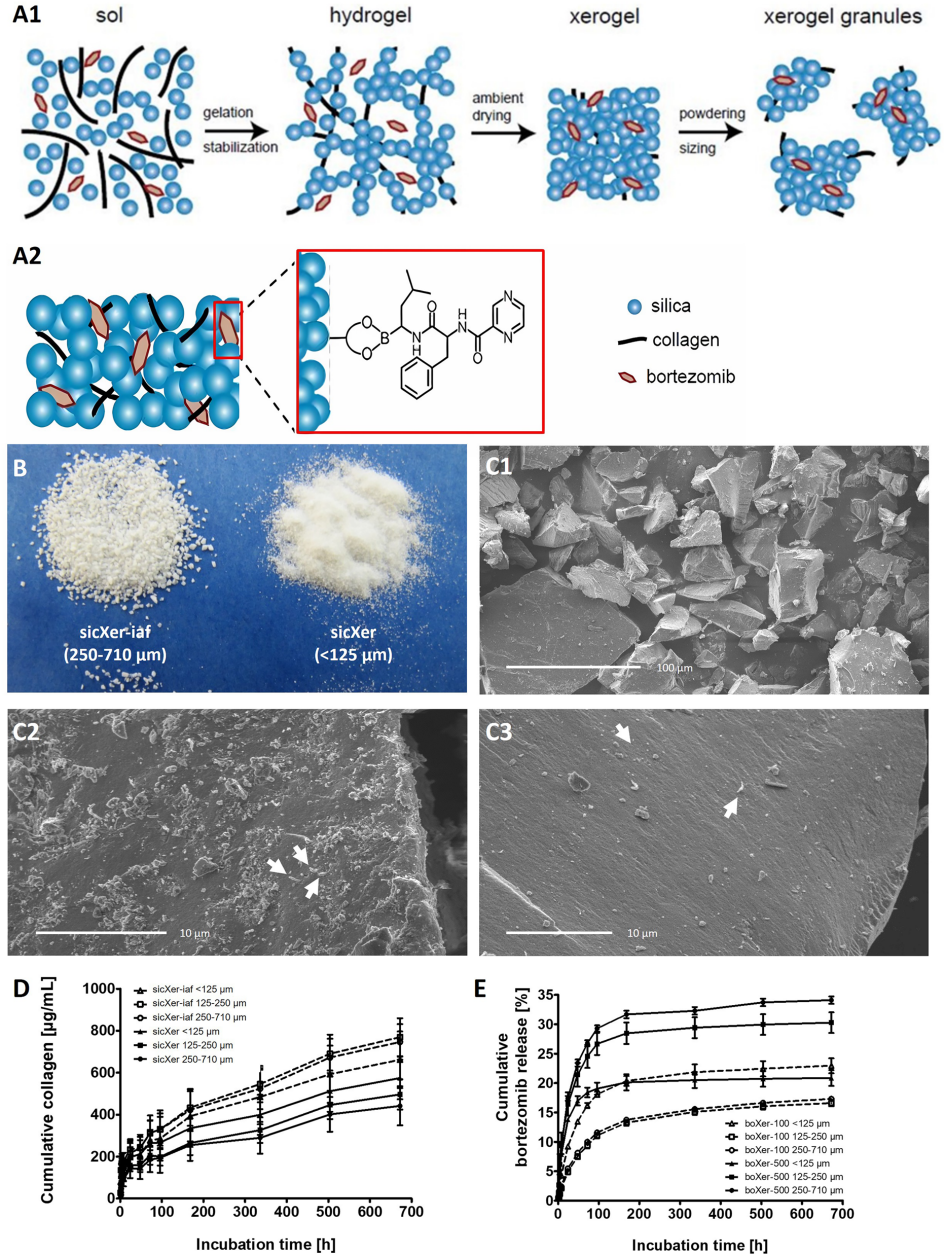
Synthesis of silica-collagen xerogels (sicXer) and bortezomib-releasing silica-collagen xerogels (boXer) as well as collagen- and bortezomib-release kinetics. **A.** Schematic view. **A1** Suspensions of bovine collagen w/wo bortezomib and silica formed a hydrogel by a sol–gel process. After drying, a mesoporous xerogel resulted. **A2** Bortezomib-binding within xerogels during the sol–gel manufacturing process, i.e., before the gel was finally formed. **B.** Photographical image of sicXer-iaf (left panel; 250–710 µm) and sicXer (right panel; < 125 µm). Bortezomib addition did not alter the visual impression (not shown). **C.** Scanning electron microscopic images of **C1** monolitic xerogels powdered to different granular sizes. **C2** Irradiation after formation (sicXer-iaf) or **C3** before formation (sicXer) can be used to impact on the form and distribution of collagen fibrils (white colored arrows) in the silica matrix. **D.** In vitro degradation of xerogel granules. Release of collagen from both sicXer-iaf and sicXer granules of different size into cell culture medium. Particle size and preparation method, i.e., irradiation before or after formation, determine in vitro collagen release from silica-collagen xerogels. **E.** In vitro bortezomib-release. Cumulative release of bortezomib from boXer-100 and boXer-500 as measured by UPLC-MS/MS related to the amount of incorporated bortezomib

SicXer and boXer synthesis is based on a sol–gel process. When mixing a buffered bovine collagen fibril suspension with prepolymerized silica, an increase in pH leads to gel formation. Drying the resulting hydrogel produces a monolithic, compact yet nanoporous composite material (Fig. 1A, B). To tailor material degradation in vivo, monolithic silica/collagen xerogels (Fig. 1C1) were grinded to granules of different sizes. Bortezomib was incorporated into silica/collagen xerogels during the sol–gel process, i.e., before the gel was formed. Activity of bortezomib was not affected by the sol–gel process, formation of xerogels, or grinding (data not shown). Different strategies were used for gamma-ray sterilization of either the final product (indicated by the suffix -iaf, e.g., sicXer-iaf, Fig. 1C2) or collagen (sicXer, Fig. 1C3). The latter strategy was introduced to increase material degradation and preclude potential gamma-ray interaction with bortezomib. Bortezomib-concertation in material is given by suffix-number as µg/g material (e.g., boxer-100, 100 µg/g bortezomib incorporated).

### Tailoring material properties

#### Mechanical properties

SicXer and boXer show primary stability comparable to trabecular bone [10, 11].

#### In vitro degradation

Granule size and preparation method (sicXer/sicXer-iaf) can tailor degradation as indicated by release of the xerogel components silica and collagen from a monolithic sample into culture medium (Fig. 1D, Supplementary Fig. S1A1, A2). Collagen-release from sicXer and sicXer-iaf (Fig. 1D) showed higher stability for sicXer vs. sicXer-iaf and a decreased release with increasing granular size, i.e., from < 125 µm to 125–250 µm to 250–710 µm for both materials as expected. After an initial burst, degradation remained constant for the incubation period (Fig. 1D). Silica release from monolithic sicXer increased during the first 16 days continuously with each medium change to 0.8 mM and remained constant thereafter between 0.6 and 1 mM (Supplementary Fig. S1A2). In parallel with degradation, culture medium is depleted of calcium and phosphate ions due to adsorptive xerogel activity (Supplementary Fig. S1A3, A4). Calcium concentration initially depleted to 0.7 mM, increased until day 7, and stayed constant at 1.3–1.4 mM thereafter. Phosphate-concentration increased first to fourfold of medium concentration with subsequently reduced release and drop to half of the initial concentration at day 5 (medium exchange on days 2, 5, and 7).

Release of bortezomib (Fig. 1E, Supplementary Fig. S1B) in cell culture medium over time from boXer-100 and boXer-500 assessed by using UPLC-MS/MS decreased from granular sizes of < 125 µm to 125–250 µm to 250–710 µm as expected. In each case, an initial burst and long-term release were observed. After 28 days of incubation, with increasing particle size, 23.9%, 16.6%, and 17.3% (boXer-100), and 20.9%, 30.3%, and 34.1% (boXer-500) of incorporated bortezomib were released.

### In vitro and ex vivo activity of sicXer and boXer

#### Bone forming and bone resorbing activity

sicXer and sicXer-iaf granules (< 125 µm) were assessed regarding impact on proliferation and osteogenic differentiation of hMSC and osteoclast differentiation.

**Fig. 2:**
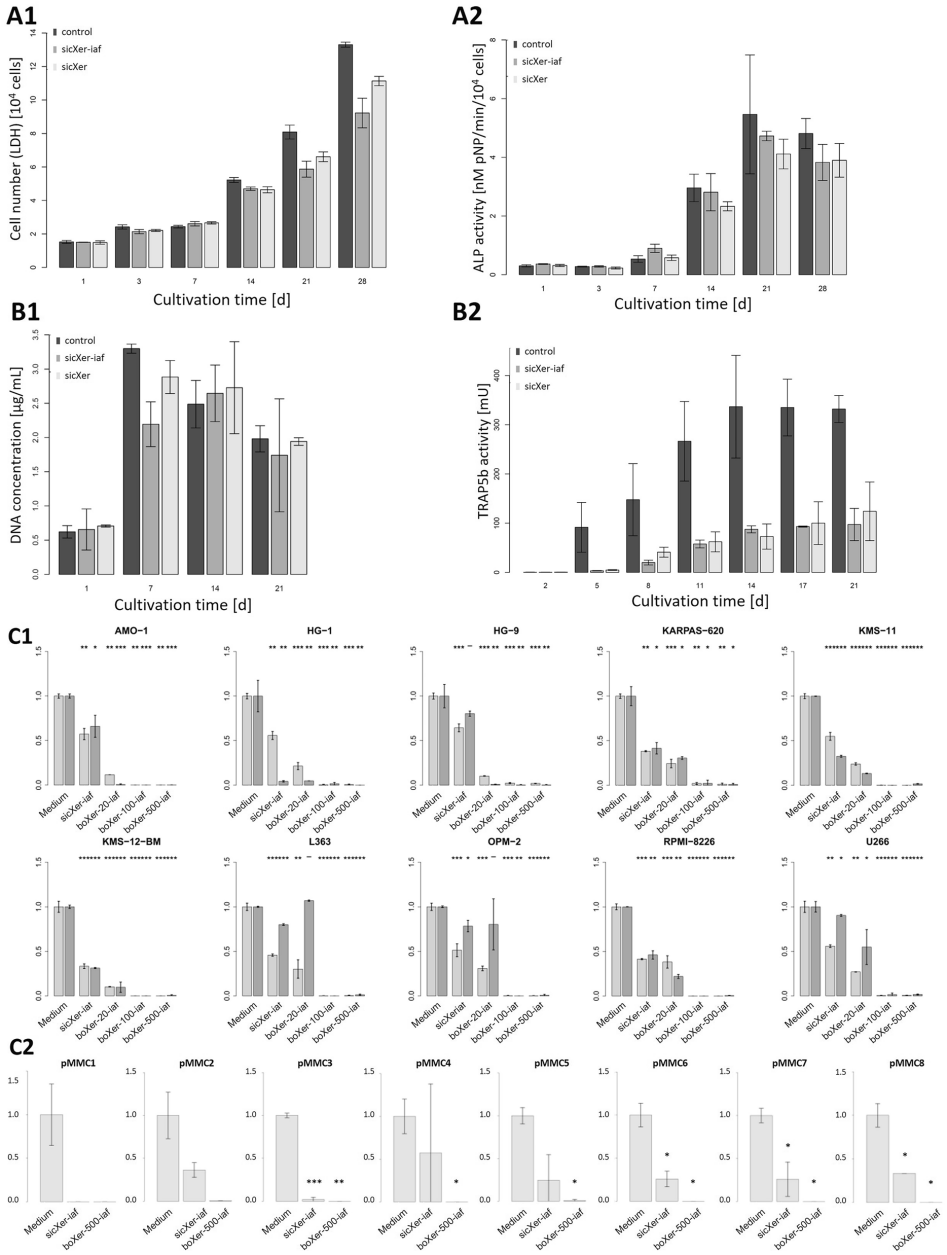
In vitro activity. sicXer and sicXer-iaf allowed increase of osteoblast number and activity over time, and significantly reduced osteoclast number and activity as compared to control, leading to net bone formation with maintained basic remodeling properties. **A1** Osteoblast formation. hMSC were cultured in absence (control), or presence of sicXer or sicXer-iaf granules of < 125 µm size under osteogenic conditions. Number of cells significantly increased over time (Jonckheere-Terpstra-test, *P* < .001). **A2** Osteoblast activity significantly increased over time as assessed by alkaline phosphatase (ALP) activity (Jonckheere-Terpstra-test, *P* < .001, all conditions). **B1** Osteoclast number and **B2** osteoclast activity. Monocytes were in vitro differentiated into osteoclasts in absence or presence of sicXer or sicXer-iaf granules with < 125 µm in size. Osteoclast activity significantly increased over time (Jonckheere-Terpstra-test, *P* < .001, all conditions) as measured by intracellular tartrate-resistant acid phosphatase type 5b (TRAP5b) activity. Osteoclast activity was significantly lower in presence of either of the bone substitute materials as compared to control. **C1** Human myeloma cell lines (n = 10) were exposed to sicXer-iaf, boXer-20-iaf, boXer-100-iaf, and boXer-500-iaf, with myeloma cell killing being visible already in the sicXer treated culture conditions. Cell culture medium was used as control. Light-grey and dark-grey colored bars represent independent experiments each performed in triplicates. **C2** Primary myeloma cells (pMMC) cultured with their bone marrow microenvironment (n = 8) were exposed to sicXer-iaf and boXer-500-iaf and compared to culture medium as control. Four of these patients (i.e., pMMC3,4,6,8) were refractory to systemic bortezomib. Please refer to Supplementary Fig. S2 for corresponding data on cells of the bone marrow microenvironment

**Osteoblast formation** was assessed by culturing hMSC in absence (control), or presence of sicXer or sicXer-iaf granules of < 125 µm size under osteogenic conditions. Number of cells significantly increased over time for all conditions (Fig. 2A1, Jonckheere-Terpstra-test, *P* < 0.001). Likewise, osteoblast activity significantly increased over time as assessed by ALP activity (Fig. 2A2, Jonckheere-Terpstra-test, *P* < 0.001, all conditions)

To assess **osteoclast formation and activity**, monocytes were in vitro differentiated into osteoclasts in absence or presence of sicXer or sicXer-iaf granules with < 125 µm in size. The number of osteoclasts increased at the beginning of the cell culture with a peak on day 7 for all conditions, followed by a decrease until day 21 (Fig. 2B1). Osteoclast activity significantly increased over time (Jonckheere-Terpstra-test, *P* < 0.001, all conditions) as measured by TRAP5b (Fig. 2B2). Osteoclast activity was significantly lower in presence of either of the bone substitute materials as compared to control.

Both materials show limited effect on osteoblast number and activity over time, but significantly reduced osteoclast activity as compared to control. The presumed resulting net bone formation with maintained basic remodeling properties was validated in vivo (see below).

#### Anti-myeloma activity

SicXer-iaf significantly induced myeloma cell killing in 10/10 human myeloma cell lines. Bortezomib-releasing materials showed an increased effect for boXer-20-iaf, with boXer-100-iaf and boXer-500-iaf almost completely eliminating remaining myeloma cells (Fig. 2C1). In primary myeloma cells obtained from bone marrow aspirates during extended myeloma diagnostic procedures [9, 39], sicXer induced significant killing. BoXer-500-iaf induced myeloma cell death > 95% in 8/8 samples. Four of these patients (pMMC3,4,6,8) were bortezomib refractory (Fig. 2C2). Toxicity on other cells of the bone marrow microenvironment was limited (Supplementary Fig. S2). Activity of both materials regarding myeloma cell killing could be validated in vivo (see below).

### In vivo activity

#### Drilling-defect in rat femora

**Fig. 3:**
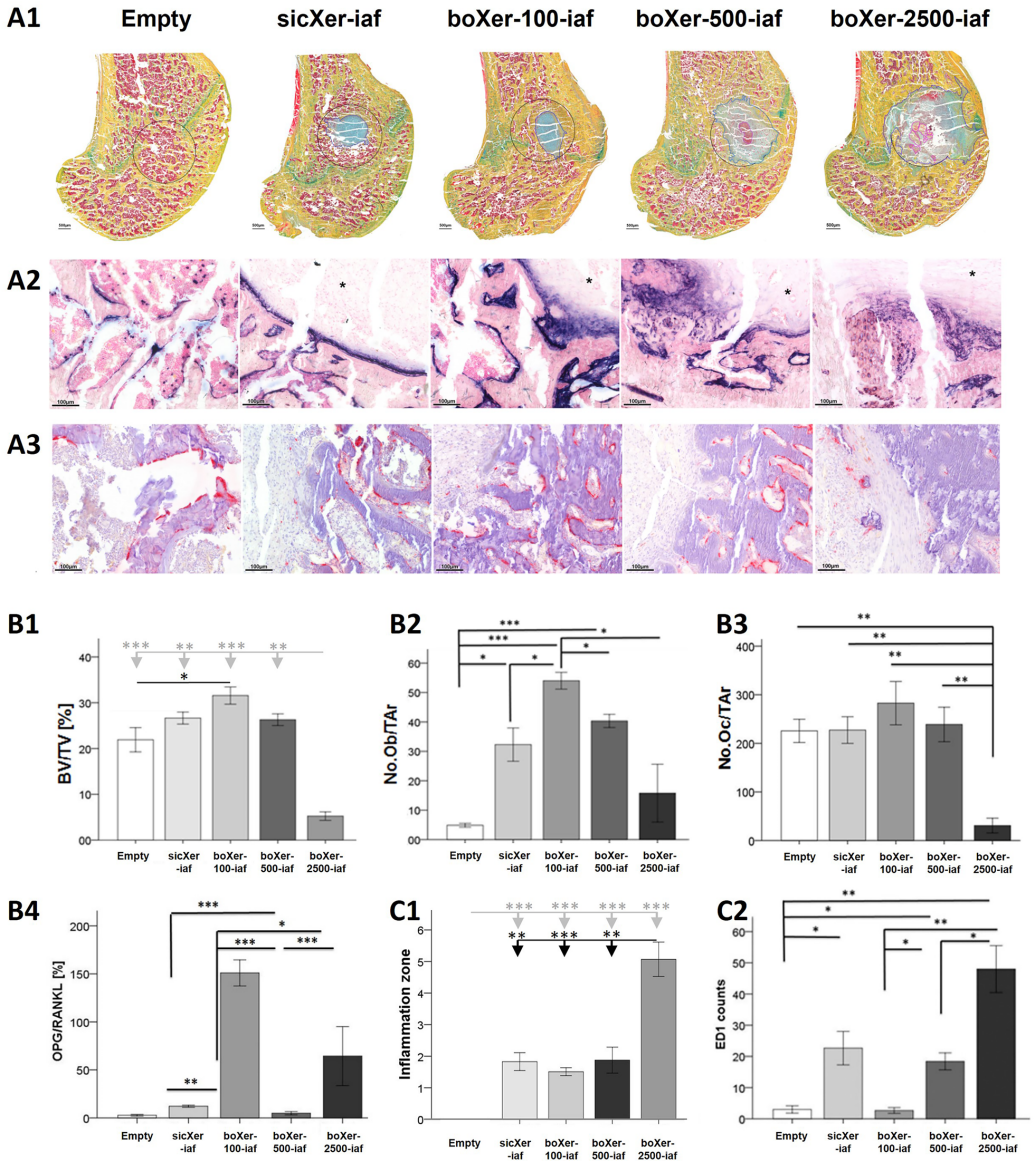

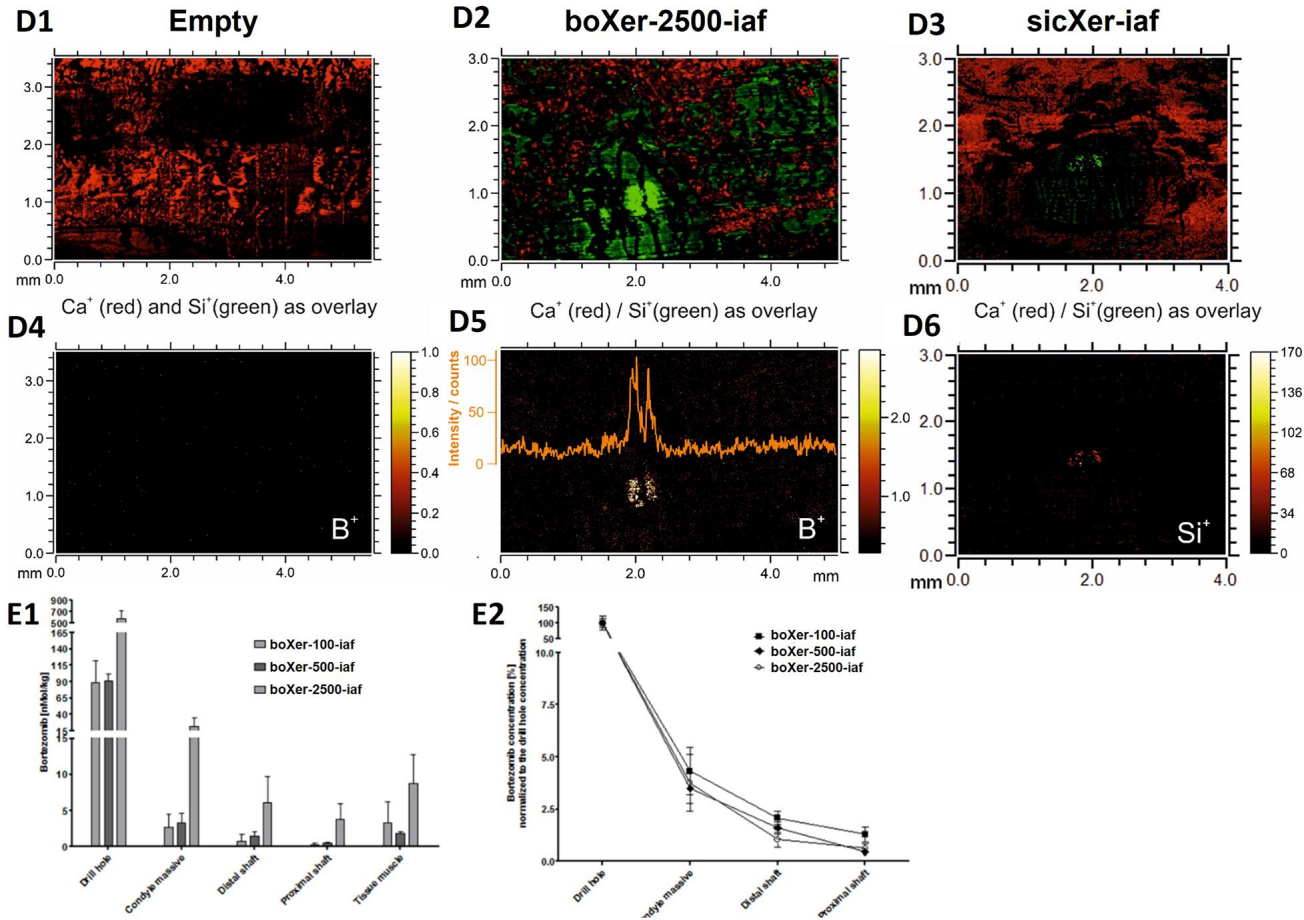
In vivo activity of sicXer-iaf and boXer-iaf in 2.5 mm drill-hole defects in healthy rats four weeks after surgery. **A1** Overview of movat pentachrome stained sections of (from left to right) empty defect, sicXer-iaf, boXer-100-iaf, boXer-500-iaf, and boXer-2500-iaf. Sections reveal enhanced new bone formation, with boXer-100-iaf exhibiting superior osteoid integration and trabecular continuity compared to controls, indicative of structural recovery similar to healthy bone. Histological analysis of **A2** alkaline phosphatase (ALP) staining showed increased ALP expression for boXer-100-iaf, consistent with active bone formation, while **A3** tartrate-resistant acid phosphatase (TRAP) demonstrated significantly fewer TRAP-positive cells in boXer-2500-iaf-treated samples (*P* = 0.002), suggesting a decrease in bone resorption. **B1** Histomorphometrical analysis of new bone formation (bone volume [BV]/tissue volume [TV]) in the initially created defect zone. Mineralized tissue was significantly higher in boXer-100-iaf treated animals when compared to empty defect (*P* = 0.038), and significantly lower for boXer-2500-iaf as compared to empty defect (*P* ≤ 0.001), sicXer-iaf (*P* = 0.002), boXer-100-iaf (*P* ≤ 0.001), and boXer-500-iaf (*P* = 0.002), respectively. Mann-Whitney test; *P < 0.05, **P < 0.01, and ***P ≤ 0.001, respectively. Grey-colored significance bars indicate comparison of boXer-2500-iaf to all other materials, black-colored in comparison to the empty control. Comparative histomorphometric analysis of **B2** number of osteoblasts (No. Ob) / trabecular area (TAr) based on ALP staining in the initially created defect zone and **B3** number of osteoclasts (No. Oc) /TAr based on TRAP staining. ALP staining showed increased expression in boXer-100-iaf. A significant decrease (*P* = 0.002) in TRAP positive cells was observed in boXer-2500-iaf treated samples. **B4** Immunohistomorphometry showed a significant increase the OPG/RANKL levels in boXer-100 when compared to all the other groups (*P* ≤ 0.001). **C1** An increase in the size of the inflammation zone as compared to the empty defect (no inflammation) was seen for sicXer, boXer-100-iaf, boXer-500-iaf, and boXer-2500-iaf (all *P* ≤ 0.001). boXer-2500-iaf showed significantly higher inflammation compared to sicXer (*P* = 0.002), boXer-100-iaf (*P* ≤ 0.001), and boXer-500-iaf (*P* = 0.004). Grey-colored significance bars indicate comparison of boXer-2500-iaf to all other materials, black-colored in comparison to the empty control. **C2** A simultaneous decrease of ED1 counts was seen in boXer-100-iaf treated animals. Corresponding immunohistochemical stainings are shown in Supplementary Fig. S3. Mann-Whitney test; *P < 0.05, **P < 0.01, and ***P ≤ 0.001, respectively. **D.** Mass spectrometric images of bone sections. **D1-D3** Mass images of the Ca^+^ and Si^+^ distribution given as overlay (Ca^+^ in red color, Si^+^ in green color) of empty defect (control), sicXer-iaf, and boXer-2500-iaf. **D4** and **D5** mass images of the B^+^ distribution. Pixels brightness correlates with count rate of B^+^. Orange line in **D5** gives the summed counts of B^+^ seen in the mass image summed up along the *y*-axis. **D6** Si^+^ image of sicXer-iaf. **E.** In vivo bortezomib concentrations in **E1** the drill hole and surrounding bone/tissue environment as well as **E2** the percentual concentration gradient from the drill hole (100%) to the condyle massive (< 5%), the distal (< 2.5%) and proximal shaft (~ 1%)

Exemplary sections stained with movat pentachrome of empty defect, sicXer-iaf, boXer-100-iaf, boXer-500-iaf, and boXer-2500-iaf reveal enhanced new bone formation, with boXer-100-iaf exhibiting superior osteoid integration and trabecular continuity compared to controls, indicative of structural recovery similar to healthy bone (Fig. 3A1). Corresponding histological analysis of ALP shows osteoblast activity, with boXer-100-iaf demonstrating increased ALP expression, consistent with active bone formation (Fig. 3A2). TRAP staining highlights osteoclast distribution and activity, with significantly fewer TRAP-positive cells observed in boXer-2500-iaf-treated samples (P = 0.002), suggesting a decrease in bone resorption (Fig. 3A3).

##### Bone formation

sicXer-iaf induced significantly increased osteoblast numbers (Fig. 3B2, *P* ≤ 0.001) at constant numbers of osteoclasts (Fig. 3B3) as compared to the empty defect and osteoblast/osteoclast activity in terms of significantly higher OPG/RANKL-ratio (*P* = 0.002; Fig. 3B4). Increased new bone formation (BV/TV) compared to the empty was observed, although failing statistical significance (Fig. 3B1).

boXer-100-iaf induced significantly increased osteoblast numbers compared to the empty defect and sicXer-iaf (P ≤ 0.001 and both *P* < 0.01, respectively), with osteoclast numbers remaining constant (Fig. 3B3). This transmitted in significantly increased bone formation (BV/TV) compared to empty (*P* = 0.038; Fig. 3B1). Expectedly, the OPG/RANKL-ratio was significantly higher compared to empty defects and sicXer-iaf (*P* ≤ 0.001 and *P* ≤ 0.001, respectively; Fig. 3B4). Higher bortezomib-concentrations diminished the positive effect on bone formation: Whereas boXer-500-iaf still significantly increased the number of osteoblasts as compared to control (*P* ≤ 0.001) at maintained osteoclast numbers, the material showed no differences in activity of osteoblast over osteoclast (OPG/RANKL) nor a significantly higher bone formation (BV/BT) as compared to the empty defect. boXer-2500-iaf did not induce a significant increase in osteoblast (Fig. 3B2) but significantly decreased osteoclast numbers (*P* < 0.01 compared to all other conditions, Fig. 3B3). For this material, bone formation (Fig. 3B1) was significantly reduced as compared to control (*P* ≤ 0.001), sicXer-iaf (*P* = 0.002), boXer-100-iaf (*P* ≤ 0.001), and boXer-500 (*P* = 0.002).

##### Tolerability

In quantitative immunohistology, an increase in size of the inflammation zone as compared to the empty defect (no inflammation) was seen for sicXer, boXer-100-iaf, boXer-500-iaf, and boXer-2500-iaf (all *P* ≤ 0.001) (Fig. 3C1). boXer-2500-iaf showed significantly higher inflammation compared to sicXer (*P* = 0.002), boXer-100-iaf (*P* ≤ 0.001), and boXer-500-iaf (*P* = 0.004). Foreign body giant cells with monocyte/macrophage phenotype expressing macrophage-associated membrane receptor CD68 (ED1; Fig. 3C2), surrogating inflammation, showed no increase for boXer-100-iaf compared to empty defect, but significantly higher values for boXer-500-iaf (*P* = 0.003) and boXer-2500-iaf (*P* ≤ 0.001).

##### Assessment of material degradation and bortezomib-distribution

Using imaging mass spectrometry (ToF–SIMS), tissue-distribution of Ca, Si, and B (Fig. 3D) was assessed. Ca-images (Fig. 3D1-3**;** red) mirrored the structure of investigated rat femora with the bone defect in the distal femur. Si (green), found not present in normal bone, was used to evaluate material degradation and distribution within bone. The majority of the sicXer-iaf and boXer-iaf is degraded after four weeks (Fig. 3D2, 3D3; Si^+^ signal in green color) with residues within the drill hole area. Released Si^+^ was distributed over the defect-surrounding tissue and accumulated in the bone marrow at the interface to the trabecula (Fig. 3D2, 3D3). Bortezomib was detectable as boron ions (B^+^) only for boXer-2500-iaf after four weeks, due to sensitivity mediated by the B-concentration and (lower) ionization potential of boron compared to Si or Ca. Main bortezomib localization was in the defect area. No concentration gradient to the neighboring tissue was seen but equal low intensity over the complete bone section (Fig. 3D5).

Bortezomib-release in vivo and tissue distribution in the operated rat femora four weeks after implantation were investigated using UPLC-MS/MS. Detectable bortezomib-concentrations depended (1) on the concentration (total amount) of the bortezomib-loading and (2) on the distance to the application site (Fig. 3E1 [absolute measurements] and Fig. 3E2 [relative measurements] normalized to bortezomib-concentration in the drill hole). Bortezomib was detectable with highest concentrations at the site of implantation in the defect of the distal metaphyseal area of the femur (69–700 nM), followed by the surrounding bone marrow of the neighboring femoral condyles (1.2–37 nM). With further distance to the defect area, bortezomib-concentration concomitantly decreased in the distal (0.1–9.5 nM) and proximal femoral (0.1–5.7 nM) shaft areas as well as in the surrounding soft tissue/muscles (0.8–13.3 nM). In all anatomical sites, the highest concentrations of bortezomib were found for the highest bortezomib-loading.

#### Drilling defects in the 5T33-myeloma mouse model

##### Load bearing and fracture stabilizing properties

No fractures occurred for either of the three used materials. Therefore, materials hindered bone fracturing by myeloma cell infiltration and stabilized the defect as intended.

##### Local control of myeloma cell infiltration

**Fig. 4:**
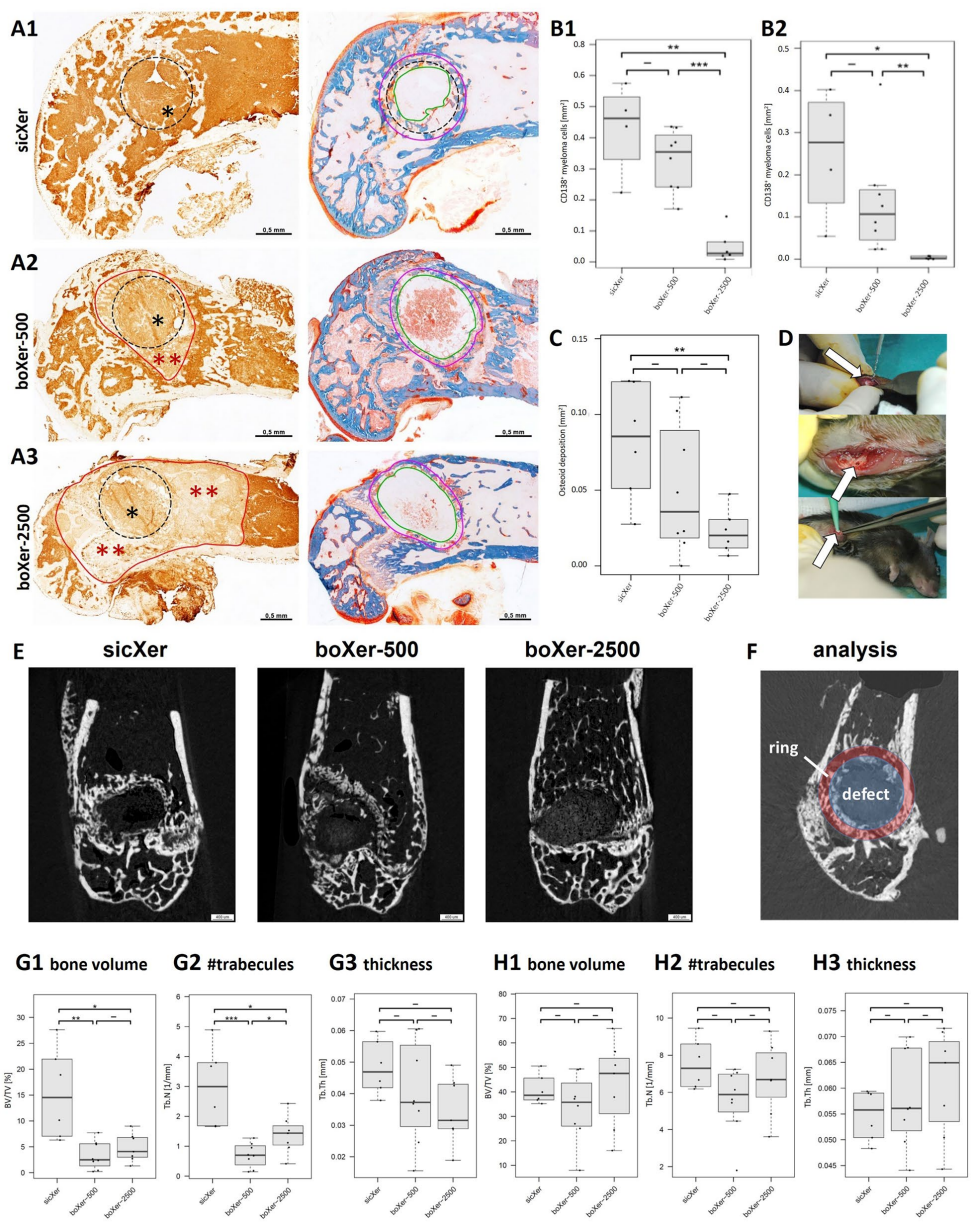
In vivo anti-myeloma and bone-formation stimulating activity of sicXer and boXer in the 5T33-myeloma mouse model. **A.** Concentration-dependent local control of myeloma cell accumulation as assessed by anti-CD138 immunohistochemistry (first column) and bone formation capacity as assessed by Masson–Goldner staining (second column) for **A1** sicXer, **A2** boXer-500, **A3** boXer-2500. After implantation of sicXer, the entire distal epiphysis was intensely stained in the course of CD138 immunohistochemistry, reflecting myeloma cell infiltration. The numerous myeloma cells even spread into the defect. After implantation of boXer-materials, bortezomib-mediated suppression effects of the myeloma cells appeared, which affected the adjacent circumference of the defect in the case of boXer-500 and extend into the diaphysis in boXer-2500. The dashed line corresponds to the contour of the drill hole defect. The red line marks the respective “limit of action of bortezomib” in terms of local control of myeloma cells. Induction of bone formation in terms of osteoid was most prominent for sicXer. As shown by Masson–Goldner staining three weeks after implantation of sicXer, woven bone has grown into the defect from the edge of the drill hole (dashed black line), while the defect areas have enlarged in the presence of boXer-materials. The green lines delimit the areas not accessed by bone, while the magenta-colored lines mark the annular zone of 100 µm around the implanted material. The osteoid margins were measured within these zones. **B.** Boxplots summarizing the results from panel **A.** in terms of reduction of myeloma cell infiltration **B1** in bone marrow and **B2** an annular zone of 100 µm around the implanted material. **C.** Boxplot summarizing the results from panel **A.** in terms of osteoid deposition. **D.** Material application in drill hole defect. White colored arrows identify the drill hole. **E.** µCT analysis of tissue slices. Coronal multiplanar reformation shows subtotal degradation of sicXer (left panel) with an enhanced bone formation inside and outside the margin of the original drillhole. BoXer-500 shows partial material resorption and new bone formation (middle panel). Almost no bone formation and material resorption were observed for boXer-2500 (right panel). **F.** Quantitative analysis of µCT-data, schematic. The defect zone represents the area of the initial bone defect (1 mm diameter, quantitative analysis in I1-I3), the “ring” represents an area of 1 mm around the initial defect (quantitative analysis in J1-J3, peri-defect area). **G1** percentage of bone in the initial defect area, **G2** number of bone trabeculae [1/mm], **G3** thickness of bone trabeculae [mm]. New bone formation in the defect is visible for all three materials, pronounced for sicXer. **H1** percentage of bone in the peri-defect area, **H2** number of bone trabeculae [1/mm], and **H3** thickness of bone trabeculae [mm]. The peri-defect zone is not negatively affected by bortezomib-release

With implanted sicXer (Fig. 4A1), myeloma cell infiltration was homogeneously present within the distal epiphysis, frequently infiltrating the drilling defect. Presence of myeloma cells in the vicinity of the drilling defect however did not hinder bone healing treated with sicXer already after three weeks (Fig. 4B2 and below). In the presence of boXer (Fig. 4A2, A3), suppression zones of myeloma cells varied in size depending on the bortezomib-concentration: the higher the bortezomib-concentration, the more pronounced the suppression. Thus, boXer-500 and boXer-2500 induced local control of myeloma cells if measured either in the whole assessed bone marrow area (Fig. 4B1), or within a 100 µm ring around the implanted material (Fig. 4B2).

##### Induction of net bone formation and bone healing

After implantation of sicXer, newly formed woven bone has grown into the defect originating from the drill edge (Fig. 4A). The defect area in histological sections was reduced from initial 0.79 mm^2^ to a mean of 0.40 (± 0.17) mm^2^, i.e., a mean of 49% of the initial defect was being covered by bone. With boXer, the contours of the drill edges were less well identifiable and the mean defect areas increase for boXer-500 to 0.86 (± 0.17) mm^2^, i.e., by a mean of 9%, and for boXer-2500 to 0.91 (± 0.14) mm^2^, i.e., by a mean of 15%. Along the “host” bone (i.e., the area surrounding the drilling defect), new bone formation was present, reflected by intensely red-colored osteoid lines that accumulate along the free edges of the trabecular bone. The respective mean osteoid areas within the ring-shaped ROI per area were 8.2 (± 3.8) % for sicXer, 4.9 (± 3.9) % for boXer-500, and 2.2 (± 1.3) for boXer-2500, Fig. 4C). For ethical reasons, no empty drill-defect controls could be tested due to frequent fractures in the 5T33-myeloma model.

Using µCT-assessment of post-mortem samples, subtotal degradation of sicXer with an enhanced bone formation inside and outside the margin of the original drillhole is found. BoXer-500 shows partial material resorption and new bone formation, while almost no bone formation and material resorption were observed for boXer-2500 (Fig. 4E,F). In the initial defect zone, mean BV/TV was 15.33 ± 8.77% for sicXer, 3.35 ± 2.94% for boXer-500, and 4.86 ± 2.83 for boXer-2500, respectively (Fig. 3G1). Thickness of trabeculae (Fig. 4G2) was 0.048 ± 0.009 µm, 0.040 ± 0.016 µm and 0.035 ± 0.011 µm for sicXer, boXer-500 and boXer-2500, respectively. Number of newly formed trabecula (Fig. 4G3) was 3.00 ± 1.32 µm^−1^, 0.70 ± 0.46 µm^−1^ and 1.39 ± 0.50 µm^−1^. In the peri-defect region, calcification was likewise visible (Fig. 4H1-3), in agreement with osteoid deposition in histological sections (Fig. 4A, C). In this region, mean BV/TV was 52.9 ± 5.7% for sicXer, 52.7 ± 9.1% for boXer-500, and 54.5 ± 19.2% for boXer-2500, respectively (Fig. 4H1). As for BV/TV, number and thickness of trabeculae did not significantly vary between the materials. The experimental procedure for the 5T33-mouse model is depicted in Fig. 4D.

Taken together, the three materials induced bone healing and prevent fractures in the 5T33-mouse model. Furthermore, they locally suppressed myeloma cell infiltration; for boXer also in the defect-surrounding tissue.

## Discussion

We present here the silica-collagen xerogels sicXer and boXer with mechanical properties comparable to trabecular bone, fostering bone formation as novel therapeutic concept for local treatment of non-malignant and malignant conditions of impaired bone healing. As part of the development process of our silica-collagen xerogels, the material has been assessed regarding mechanical properties, including stability: studies on monolithic bone substitutes showed these to withstand the mechanical stresses encountered during implantation into bone. In the wet state, the elastic modulus and their compressive as well as tensile strengths ranged between the reference values ​​for human spongiosa and cortical bone [16, 17]. In this manuscript, we show that intendedly both materials stabilized the defects in all animals (rats and mice) without any observed fractures.

Presumed mediators of material activity are first presence and release of silicon, shown, e.g., for calcium phosphate cements increasing in vitro osteoblastogenesis [40] and in vivo osteogenesis [41], or silicon-based (bioceramic) scaffolds [42, 43]. Secondly, adsorption to silica-xerogels removes elements of intercellular bone-remodeling crosstalk in the implant material’s vicinity [44]. Previous studies found bortezomib to stimulate bone formation by fostering osteoblast development and function at concentrations of ≈5 nM [22, 23], and to repress bone resorption, including induction of osteoclast apoptosis [22, 23, 45–48] (IC_50_≈92 nM) [49]. This relates well to bortezomib-concentrations measured in the defect’s vicinity (rat model, ≈37 nM after four weeks), explaining increased bone healing by boXer as compared to sicXer. Mechanistically, in the rat model, both materials shift the OPG/RANKL-pathway to bone formation by simultaneous increase of OPG- and decrease of RANKL-levels with concomitant increase of osteoblast number and bone mass. This effect is strongest for low bortezomib-containing materials, i.e., boXer-100-iaf. Higher bortezomib-release by boXer-500-iaf diminishes this effect, and the highest release by boXer-2500-iaf leads to a strong inflammation reaction. In the 5T33-model, bone formation is visible by osteoid deposition and calcification in the peri-defect region, especially for boXer-500, but not in the defect per se, as the duration of the 5T33-model needed to be limited as mice had to be euthanized due to development of systemic multiple myeloma. Compared with the rat model, the 5T33-model thus gives an earlier snapshot of the bone healing process. In vivo induction of bone healing is in agreement with a recent study in a rabbit femoral defect model using bortezomib‑loaded porous nano‑hydroxyapatite/alginate scaffolds [50]. In contrast to bortezomib-releasing polymethylmethacrylate [51], boXer and sicXer allow remodeling of the defect to bone.

In multiple myeloma, accumulation of malignant plasma cells impacts bone remodeling. After an initial increase of both bone resorption and bone formation, the latter cannot hold pace and a local loss of net bone mass results [52], leading to osteolytic lesions. Myeloma cells, depending on auto- and paracrine growth and survival factors like insulin-like growth factor 1 [28, 53–55], profit from their release from the bone marrow environment and degraded bone matrix, constituting a vicious cycle. Elimination of myeloma cells removes the primary stimulus and allows bone to heal.

Anti-myeloma activity against human myeloma cell lines and primary myeloma cells is already induced by sicXer. As normal and malignant myeloma cells are dependent on the presence of growth and survival factors (see e.g., review in [53]), we hypothesize that silica-collagen xerogels which are known to easily absorb [21, 44] deprive the myeloma cell population of these factors, which at least in part mediates myeloma cell death. For boXer, killing by bortezomib strongly increases this effect. Measured local bortezomib tissue concentrations surpass the IC_50_-value of myeloma cell lines (1.6–8.8 nM) [56], including those made bortezomib-resistant with 20-fold higher IC_50_ [57], explaining fostered boXer activity also in primary myeloma cells from bortezomib-resistant patients. In the 5T33-mouse model, the area surrounding the defect is also cleared from myeloma cells.

**Fig. 5:**
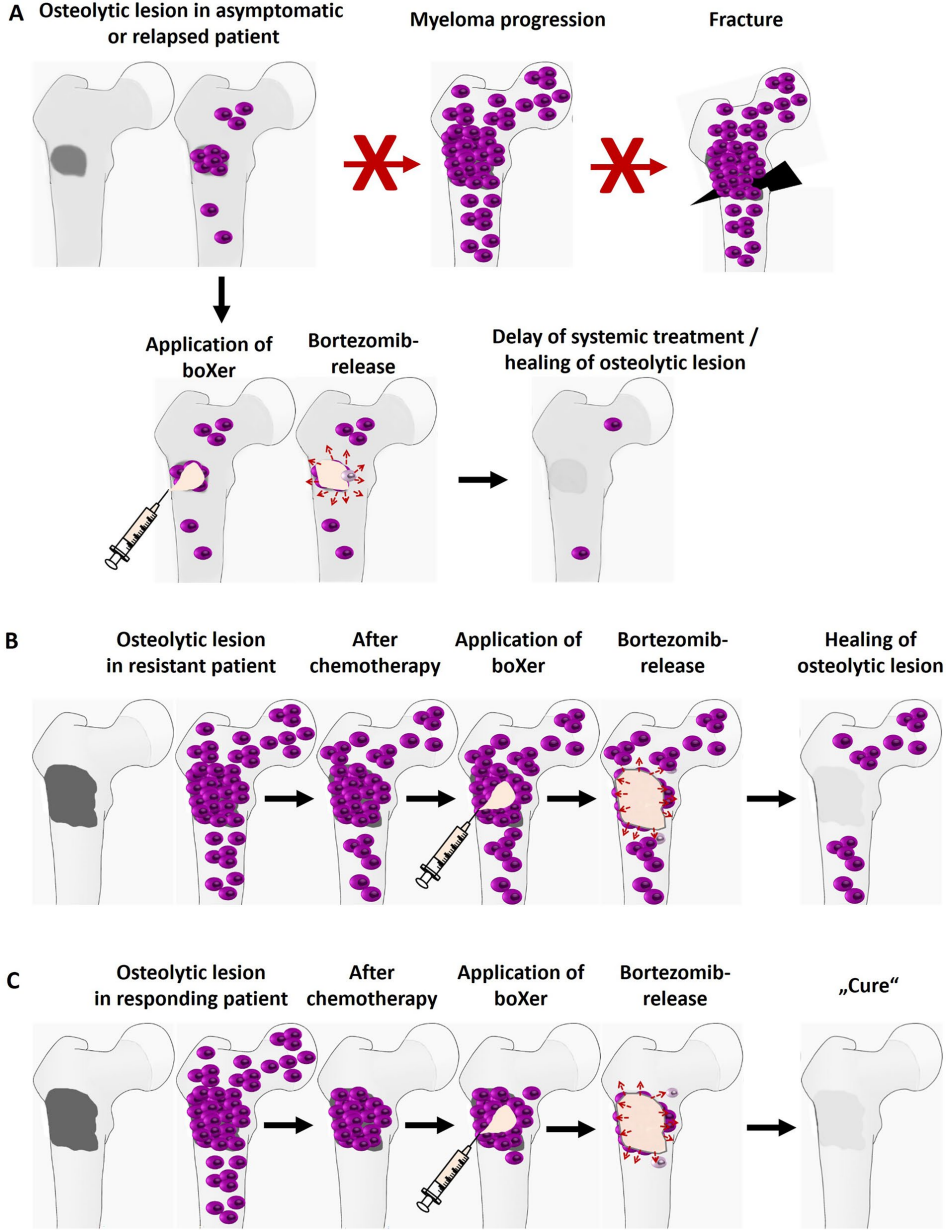
Potential applications of sicXer and boXer in multiple myeloma treatment. **A.** In asymptomatic or relapsed myeloma, progressing osteolytic bone lesions are a frequent reason to start treatment to avoid pathological fractures and major structural damage. **B.** A specific condition is present if this appears in a patient either resistant to all applicable treatments, or in whom, due to side effects of previous treatment, no more systemic chemotherapy or radiotherapy can be applied. Local release of high bortezomib doses (e.g., using boXer-500) could, due to applicable doses, overcome bortezomib-resistance and lead to local tumor control and, with the mechanical properties of the material, to stabilization. **C.** In the absence of minimal residual disease, osteolytic lesions represent potential “safe zones” in which myeloma cells have survived treatment. Eliminating these let envision prolonged time to progression or even, optimistically, can contribute to a cure of myeloma

sicXer and boXer could be used for treatment of fractured or fracture-prone osteolytic lesions (Fig. 5), stabilizing the defect and locally controlling malignant plasma cells, even if these are resistant against systemically achievable bortezomib-concentrations. Unlike percutaneous radiotherapy, locally applied biomaterials directly improve biomechanical defect-stability, stimulate bone healing, and do not impact surrounding (hematopoietic) tissue. In either asymptomatic (smoldering) myeloma patients, where the later occurrence of osteolytic bone lesions is the most frequent and in one third of patients only indication for systemic chemotherapy [4], and a subgroup of successfully systemically treated patients, in which disease progression or relapse *only* manifests in growing lytic lesions, local fracture-preventing treatment could act to delay or bridge to (further) systemic treatment (Fig. 5). Given that osteolytic lesions are frequently the last sites in which myeloma cells can be detected as surviving systemic treatment [5, 6], eliminating these “holdouts” could be part of a combined systemic/local treatment strategy in clinical trials. In non-malignant conditions, both materials could be indicated; boXer because of higher bone-anabolic properties and limited effect beyond the immediate defect area.

While the highest bortezomib-dose in our experimental setting (boXer-2500) was primarily not intended for clinical use, but to study a potentially toxic bortezomib effect in the assessment of a “sweet-spot”, the materials presented here, i.e., sicXer, boXer-100 (“low dose bortezomib”), and boXer-500 (“high dose bortezomib”), would allow adapting treatment to situations in which a particular aspect of material activity would be especially warranted. I.e., sicXer or boXer-100 if the focus lies on stabilization of potentially fracture-prone lesions, and higher bortezomib-release if focusing on control of (residual) myeloma cells. Evidently, this concept would need to be tested within a clinical trial setting.

## Conclusions

In summary, we present here sicXer and boXer as novel therapeutic concept. The combination of stabilization of fracture-prone lesions, stimulation of bone healing, and anti-tumor effect suggest clinical testing of sicXer and boXer as part of a combined systemic/local treatment strategy in multiple myeloma and non-malignant diseases.

## Supplementary Information

Additional file 1.

## Data Availability

All data generated or analyzed during this study are included in this published article and its supplementary information file.
