# Correction: Bortezomib-releasing silica-collagen xerogels for local treatment of osteolytic bone- and minimal residual disease in multiple myeloma

**DOI:** 10.1186/s13045-025-01663-9

**Published:** 2025-01-24

**Authors:** Dirk Hose, Seemun Ray, Sina Rößler, Ulrich Thormann, Reinhard Schnettler, Kim de Veirman, Thaqif El Khassawna, Christian Heiss, Anne Hild, Daniel Zahner, Francisca Alagboso, Anja Henss, Susanne Beck, Martina Emde-Rajaratnam, Jürgen Burhenne, Juliane Bamberger, Eline Menu, Elke de Bruyne, Michael Gelinsky, Marian Kampschulte, Marcus Rohnke, Sabine Wenisch, Karin Vanderkerken, Thomas Hanke, Anja Seckinger, Volker Alt

**Affiliations:** 1 Laboratory of Hematology and Immunology & Labor für Myelomforschung, Vrije Universiteit Brussel, Laarbeeklaan 103, Jette, 1090 Belgium; 2 Experimentelle Unfallchirurgie (ForMED), Justus-Liebig-Universität Gießen, Aulweg 128, 35392 Gießen, Germany; 3 Institut für Werkstoffwissenschaft, Max-Bergmann-Zentrum für Biomaterialien, Technische Universität Dresden, Budapester Straße 27, 01069 Dresden, Germany; 4 Justus-Liebig-Universität Gießen, Ludwigstraße 23, 35392 Gießen, Germany; 5 Klinische Anatomie und Experimentelle Chirurgie C/O Institut für Veterinär-Anatomie, -Histologie und - Embryologie, Justus-Liebig-Universität Gießen, Frankfurter Straße 98, 35392 Gießen, Germany; 6 I. Physikalisches Institut, Justus-Liebig-Universität Gießen, Heinrich-Buff-Ring 16, 35392 Gießen, Germany; 7 Innere Medizin IX - Abteilung für Klinische Pharmakologie und Pharmakoepidemiologie, Medizinische Fakultät/Universitätsklinikum Heidelberg, Im Neuenheimer Feld 410, 69120 Heidelberg, Germany; 8 Labor Für Experimentelle Radiologie, Justus-Liebig-Universität Gießen, Carl-Maria-von-Weber-Straße 8, 35392 Gießen, Germany; 9 Zentrum für Translationale Knochen-, Gelenk- und Weichgewebeforschung, Technische Universität Dresden, Fetscherstraße 74, 01307 Dresden, Germany; 10 Physikalisch-Chemisches Institut, Justus-Liebig-Universität Gießen, Heinrich-Buff-Ring 17, 35392 Gießen, Germany; 11 Klinik und Poliklinik für Unfallchirurgie, Universitätsklinikum Regensburg, Franz-Josef-Strauß-Allee 11, 93053 Regensburg, Germany

**Journal of Hematology & Oncology (2024) 17:128**

10.1186/s13045-024-01636-4

The original article mistakenly omitted last author, Volker Alt as a co-Corresponding Author due to an error mistakenly carried forward by the production team.

Dr. Alt has since been restored as co-Corresponding Author.

