# Supplementary material for: Bortezomib-releasing silica-collagen xerogels for local treatment of osteolytic bone- and minimal residual disease in multiple myeloma
Source: J Hematol Oncol. 2024 Dec 18;17:128. doi: 10.1186/s13045-024-01636-4 (PMC11657678; doi:10.1186/s13045-024-01636-4)
Supplement: Supplementary file 1 — Additional file 1. [file 13045_2024_1636_MOESM1_ESM.pdf]

SUPPLEMENTARY APPENDIX

SUPPLEMENTARY FIGURES

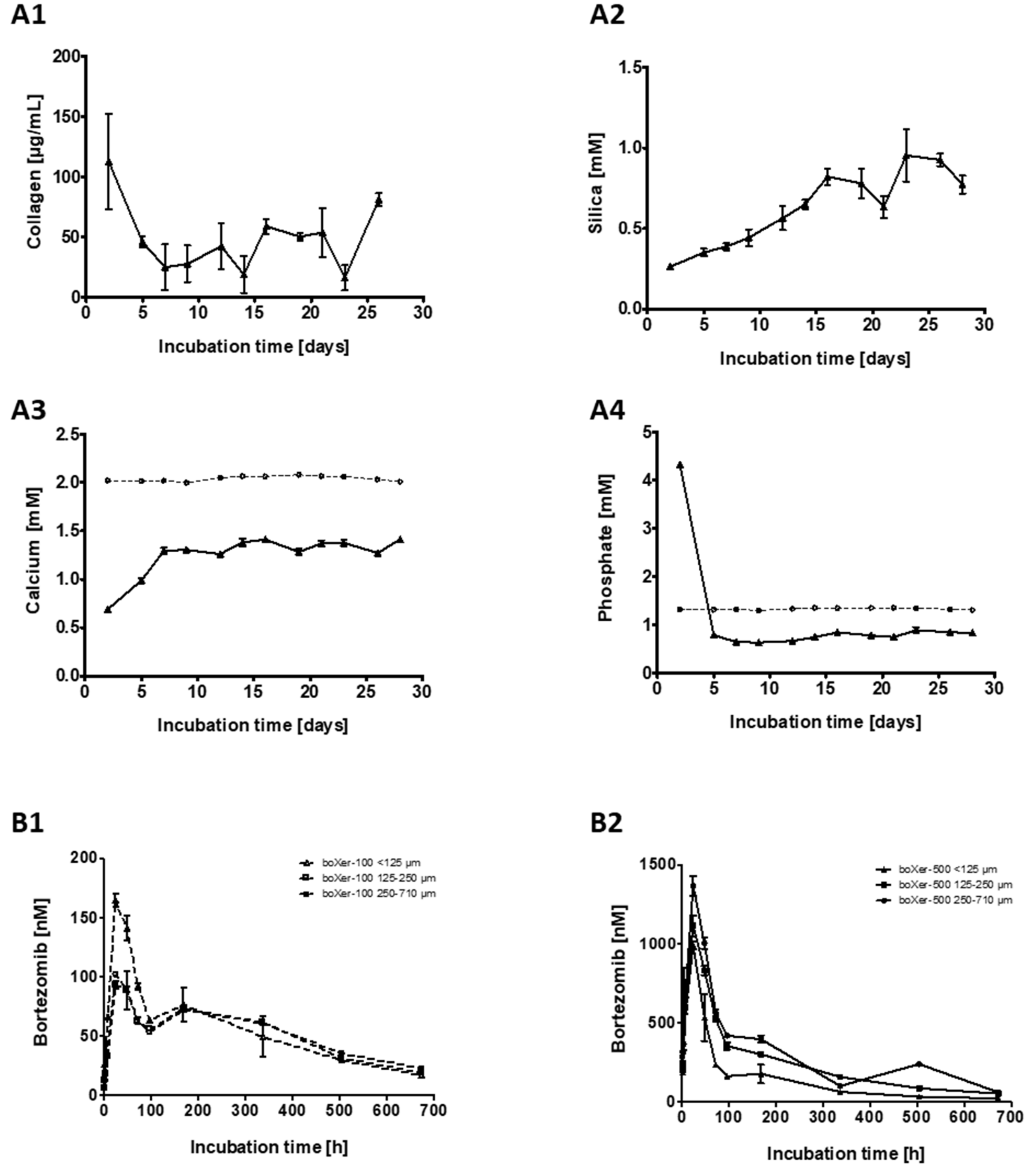

<u>Supplementary Figure S1</u>. ***In vivo* and *in vitro* material degradation kinetics and bortezomib-release. A.** *In vitro* degradation of sicXer. Release of **A1** collagen and **A2** silica from monolithic sicXer into cell culture medium (dotted line). Change in medium **A3** calcium and **A4** phosphate ion concentration. **B.** *In vitro*

bortezomib-release from boXer. Release of bortezomib from boXer granules of different size, for **B1** boXer-100 and **B2** boXer-500. Bortezomib-concentration was measured by UPLC-MS/MS. Experiments were performed in triplicates.

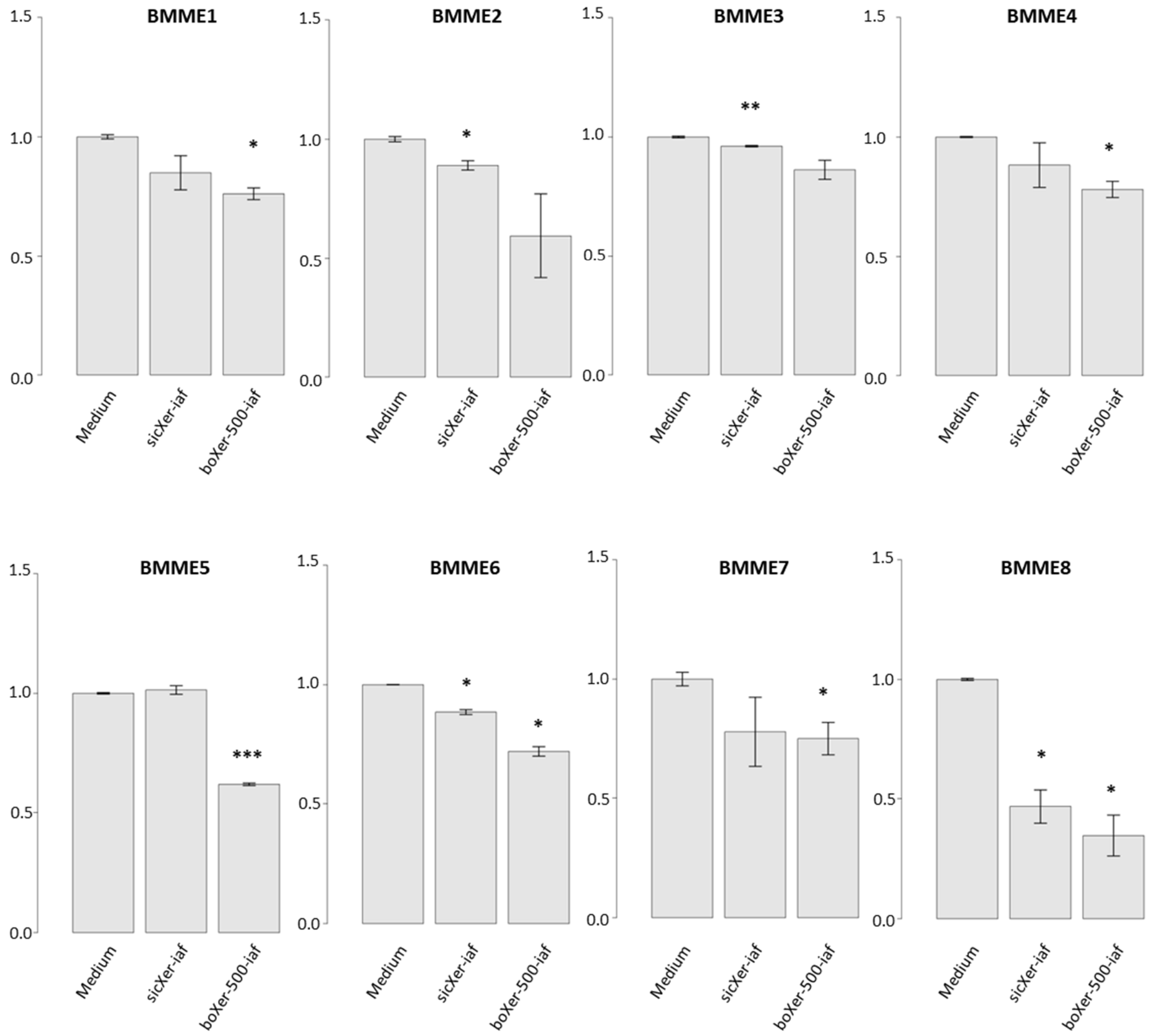

<u>Supplementary Figure S2</u>. Anti-myeloma activity of sicXer and boXer on cells of the bone marrow microenvironment (BMME). Primary cells from patients with multiple myeloma were exposed to sicXer-iaf, boxer-500-iaf, and boxer-2500-iaf, respectively. Samples and description complementary to Fig. 2.

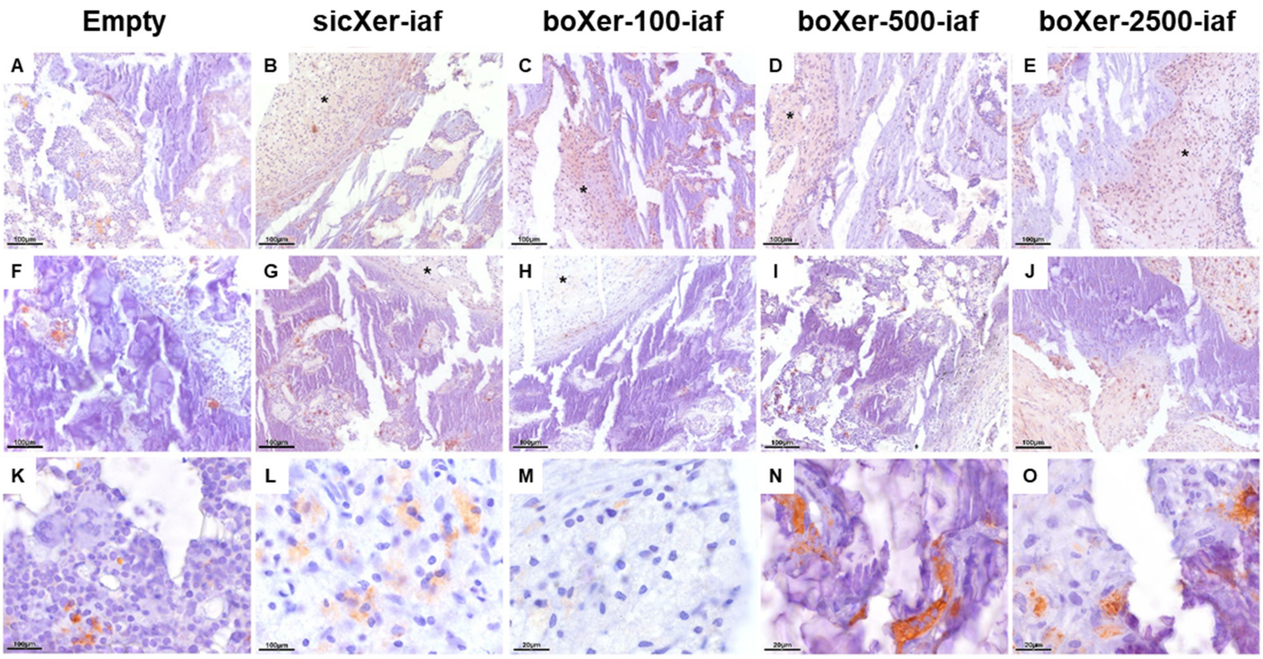

__Supplementary Figure S3__**. Activity of sicXer-iaf and boXer-iaf in 2.5 mm drill-hole defects in healthy rats four weeks after surgery.** Immunhistochemical stainings of **A-E** osteoprotegerin (OPG), **F-J** receptor activator of NF-κB ligand (RANKL), and **K-O** CD68 (ED1) in empty defect, sicXer-iaf, boXer-100-iaf, boXer-500-iaf, and boXer-2500-iaf (from left to right), respectively. Please see Fig. 3 for additional information.
